# Dealloying of Cu-Based Metallic Glasses in Acidic Solutions: Products and Energy Storage Applications

**DOI:** 10.3390/nano5020697

**Published:** 2015-04-29

**Authors:** Zhifeng Wang, Jiangyun Liu, Chunling Qin, Hui Yu, Xingchuan Xia, Chaoyang Wang, Yanshan Zhang, Qingfeng Hu, Weimin Zhao

**Affiliations:** 1 School of Materials Science and Engineering, Hebei University of Technology, Tianjin 300130, China; E-Mails: wangzf@hebut.edu.cn (Z.W.); liujiangyun1991@163.com (J.L.); yuhuidavid@hebut.edu.cn (H.Y.); xc_xia@hebut.edu.cn (X.X.); wangchaoyang92@126.com (C.W.); 15222812590@163.com (Y.Z.); huqingfeng_hebut@163.com (Q.H.); 2 Key Laboratory for New Type of Functional Materials in Hebei Province, Hebei University of Technology, Tianjin 300130, China; 3 CITIC Dicastal Co. Ltd., Qinhuangdao 066011, China

**Keywords:** dealloying, metallic glass, nanoporous copper, energy storage, application

## Abstract

Dealloying, a famous ancient etching technique, was used to produce nanoporous metals decades ago. With the development of dealloying techniques and theories, various interesting dealloying products including nanoporous metals/alloys, metal oxides and composites, which exhibit excellent catalytic, optical and sensing performance, have been developed in recent years. As a result, the research on dealloying products is of great importance for developing new materials with superior physical and chemical properties. In this paper, typical dealloying products from Cu-based metallic glasses after dealloying in hydrofluoric acid and hydrochloric acid solutions are summarized. Several potential application fields of these dealloying products are discussed. A promising application of nanoporous Cu (NPC) and NPC-contained composites related to the energy storage field is introduced. It is expected that more promising dealloying products could be developed for practical energy storage applications.

## 1. Introduction

Dealloying, which is a well known etching technique, refers to selective dissolution of one or more components out of an alloy [1], leaving residual noble metal nanoporous structure. Such a technique, initially known as depletion gilding [2], has been used by metalsmiths to gold-coat artifacts for millennia, dating back to the Nagada period (prehistory 4000–3100 BC) [2]. However, some speculate that a nanoporous structure is formed during alloy corrosion owing to the limitation of observation at the nanoscale in the past. This idea has recently been receiving renewed attention because various dealloying products, including nanoporous metals, nanoporous alloys, metallic oxide and composites with excellent physical and chemical properties, can be fabricated by this method [3,4,5,6] and can be clearly observed using a high performance scanning electron microscope (SEM) and transmission electron microscopy (TEM), in recent decades [7]. As a result, research on products and applications of an alloy after dealloying has become a hot topic and is drawing increasing attention.

Nanoporous metals and alloys are known as easily obtained dealloying products after corrosion. With the development of dealloying techniques and theories, nanoporous metals such as Au [8], Pd [9], Pt [10], Ag [11] and Cu [12], as well as nanoporous alloys including Au–Ag [13], Pt–Ru [14], Pd–Ag [15] and Pt–Au [16] have been developed in the latest 15 years. They have been revealed for potential applications in catalysis [17], heat exchangers [18], actuators [19], energy storage [20], biosensors [21] and surface-enhanced Raman scattering [22]. Besides this applied research, some basic research related to dealloying has been carried out. Erlebacher and coworkers [23] proposed an atomistic model to explain the formation mechanism of the nanoporous structure during dealloying. Delogu’s group [24] developed theoretical models to relate structure and mechanical properties in nanoporous metals. Hosson *et al.* [25,26] studied strain in nanoporous gold. Biener [27], Lian [28] and Weissmüller *et al.* [29] studied mechanical properties of nanoporous metal and composites. It was found that after mixing nanoporous gold with epoxy, the composite became stronger and harder than each constituent phase individually. Thus, as a kind of engineering material, the application field of nanoporous metal can be extended. However, extensive studies have focused on the fabrication and properties of nanoporous noble metals and alloys. Research pertaining to inexpensive nanoporous metals and other fascinating dealloying products is quite rare. As we know, the cost must be taken into account for practical commercial application of the dealloyed products. Recently, a new series of dealloyed products including nanoporous Cu (NPC) and nano/micro-Cu oxides with low cost and unique properties have been developed in our group [30,31,32,33,34]. This paper presents an overview of our recent advancement of dealloyed products via free-dealloying Cu-based metallic glasses (MGs) in hydrofluoric acid (HF) and hydrochloric acid (HCl) solutions, as well as a brief review of others’ results related to dealloying. The prospective potential applications of the dealloyed NPC and composites are also introduced.

## 2. Fabrication of NPC

The fabrication of inexpensive NPC has attracted the attention of numerous researchers. Many direct factors, such as alloy composition, dealloying solutions, etching time, dealloying temperatures and electrochemical potential, have been widely studied [35,36,37,38,39]. These factors are confirmed to influence the specific structural characteristics of the NPC.

**Figure 1:**
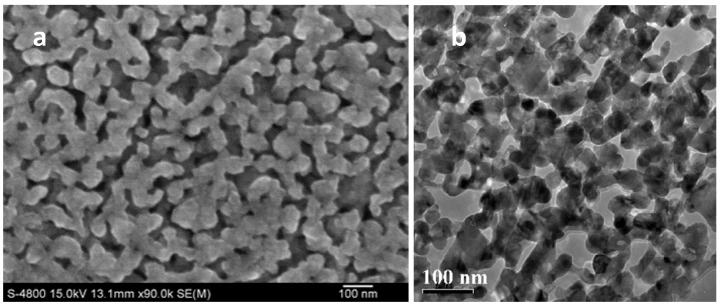
Typical nanoporous Cu (NPC) obtained by dealloying of Cu_52.5_Hf_40_Al_7.5_ MGs in 0.5 M hydrofluoric acid (HF) solution for 300 s [30]. (**a**) Scanning electron microscope (SEM) image; (**b**) Transmission electron microscopy (TEM) image.

Based on a large number of experiments, the microstructure and chemical composition of a precursor alloy are the key parameters to determine the dealloying process and the morphology of nanopores. To prepare uniform nanoporous metal, a homogeneous single phase of dealloying precursor is necessary. Due to their single phase nature, representative single phase solid solution and metallic glasses are good candidates as dealloying precursors. For example, Au–Ag alloy [40] is an ideal and classical precursor to produce uniform nanoporous Au by selectively dissolving the constituent element Ag, because Au and Ag are completely miscible through the entire composition range and no phase separation occurs during the dealloying process. For two-phase or multiphase alloy systems, however, the dealloying results are complicated. In the case of a two-phase system, if one phase can be removed from the alloy and another remains, porous/nonporous metal composites can be fabricated. If the dealloying takes place within each phase, then nanoporous metal composite with two kinds of nanoporous metals or two different pore size distributions can be obtained [41,42]. Up to now, Mn–Cu [43], Al–Cu [44], Mg–Cu [45] and Zn–Cu [46] crystalline precursors have been used to produce NPC with different porous structures. Aside from crystal precursors, recently, amorphous materials such as MGs have also been demonstrated to be a new type of precursor for dealloying to nanoporous metals. MGs are known for their chemically homogeneous single-phase nature which lack crystalline defects and large-scaled phase segregations. They have thousands of compositions and can be used to fabricate various nanoporous metals which cannot be achieved from conventional crystalline precursors. Nowadays, MG precursors including Ti–Cu(–Au) [47,48], Mg–Cu–Y [49], Mg–Cu–Gd [50], Cu–Zr–Ti [51], Cu–Hf–Al [30], Cu–Zr(–Al) [31] and Al–Cu–Mg(–Ni) [52] have been used to obtain uniform NPC through chemical/electrochemical dealloying processes. As shown in Figure 1, a NPC ribbon, which obtained by dealloying of Cu–Hf–Al MGs [30], presented a typical three-dimensional (3D) continuous nanoporous structure. For a binary MG, different atomic ratios influence the pore size of the resulting 3D nanoporous structure. For example, as atomic ratios of the Cu in Ti–Cu ribbons increased, the pore size of NPC decreased [47]. Moreover, the ligament/pore size of dealloyed NPC can be tailored by the addition of different third components into binary MGs. For instance, the pore size of NPC dealloyed from the Ti_60_Cu_39_Ag_1_ alloy was smaller than that of the Ti_60_Cu_40_ alloy [53]. A noble Au additive in Ti–Cu MGs resulted in the formation of an NPC with ultrafine nanoporous structure [48]. An active Al additive in Cu–Zr MGs, however, led to the formation of a wider ligament of dealloyed NPC [31].

**Figure 2:**
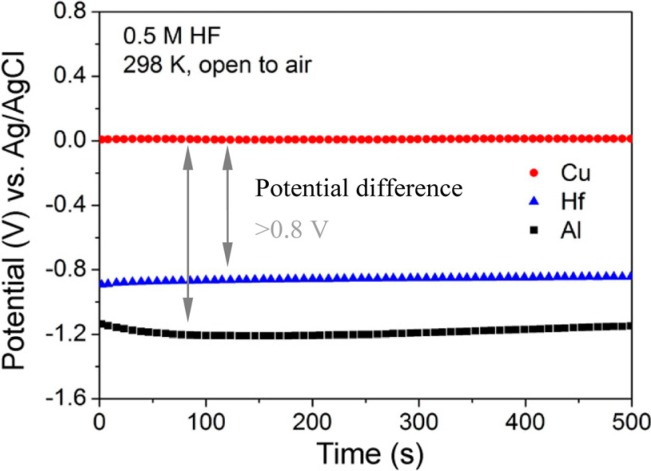
Open-circuit potentials of metals Cu, Hf and Al in 0.5 M HF solution at 298 K open to air [30].

For dealloying study, a proper etching solution must be correctly selected. For obtaining NPC using a dealloying method, the constituent elements of the precursor alloy exhibiting a large difference in the galvanic series [54] in an etching solution is a necessary requirement. Figure 2 shows open-circuit potentials *vs.* time curves of the metal Cu, Hf and Al in 0.5 M HF solution at 298 K open to air. Among the constituent elements, Cu metal shows much higher stability in HF solution due to its much nobler potential, whereas Hf and Al metals exhibit high electrochemical activity. It can be also seen from Figure 2 that the potential differences between Cu and other two elements are noticeable and greater than 0.8 V, which provides an advantageous driving force for the dissolution of less noble elements Hf and Al under free corrosion conditions. As a result, a uniform NPC can be obtained by dealloying the Cu_52.5_Hf_40_Al_7.5_ MG in 0.5 M HF solution [30]. Various corrosive solutions, such as HCl [55], H_2_SO_4_ [56], HF [57], H_3_PO_4_ [58] and HBF_4_ [59] solutions, NaOH aqueous alkali [60] and NaCl saline solution [61], were successfully used to produce NPC. Representative NPC obtained by dealloying of MGs in different electrolytes is listed in Table 1.

**Table 1:** Representative NPC obtained via dealloying of MGs.

Dealloying Solution	Precursors	References
HCl	Al_70_Cu_18_Mg_12_, (Al_75_Cu_17_Mg_8_)_97_Ni_3_	[52,62]
H_2_SO_4_	Mg_90−*x*_Cu*_x_*Y_10_ (*x* = 20, 25, 30, 40 at.%)	[49,56]
	Mg_65_Cu_25_Gd_10_	[50]
H_2_SO_4_ + PVP	Mg_65_Cu_25_Y_10_	[12]
HF	Cu_52.5_Hf_40_Al_7.5_	[30]
	Cu_50_Zr_50−*x*_Al*_x_* (*x* = 0, 5 at.%)	[31]
	Cu_60_Zr_30_Ti_10_	[51]
	Ti_100−*x*_Cu*_x_* (*x* = 33, 40, 50, 60, 70 at.%)	[47,63,64]
	Ti–Cu–(Ag, Au, Ni, Pd, Pt)	[48,53,57,65,66,67]
HF + PVP	Ti_60_Cu_40_	[68]

Diverse acidic solutions have been widely used for dealloying to produce NPC. It was reported that the ligament/pore size of dealloyed NPC was strongly influenced by the corrosive solution. Generally, the length scale of the NPC ligament and pore size turned to be greater in more concentrated solutions [47]. In addition, an introduction of organic macromolecules into dealloying solution was conducive to refined nanoporous structures [12,68]. It was reported that the pore and ligament sizes decreased in a mixed solution of poly-vinylpyrrolidone (PVP) and sulfuric acid compared to those in the PVP-free H_2_SO_4_ solution. With increasing concentration of PVP in the dealloying solutions, the sizes of nanopores and ligaments decreased.

Besides the precursor composition and dealloying solution, etching conditions such as dealloying time and temperature also have great effects on the ligament/pore size of the nanoporous metals. Controlling etching conditions is a simple and effective way to tailor porous structures, which determines the final physical and chemical properties of the NPC. Since the dealloying takes place layer by layer, the thickness of the nanoporous metal increases with increasing the etching time. By choosing a proper etching time, we can produce a complete 3D nanoporous structure material without further coarsening of the ligament/pore. Meanwhile, inefficient dealloying of a MG precursor resulted in the fabrication of a nanoporous metal/MG/nanoporous metal composite with a sandwich-like structure [30]. The sandwich-like structure composites containing a MG ductile interlayer showed good bendability [30]. It was presented that the interplay between ligament width *d* of NPC and dealloying time *t* can be deduced [56] as:

(1) $$\ln d=\frac{1}{n}\ln t-\frac{E}{nRT}+\frac{1}{n}\ln(KD_{0})$$

where *n* is the coarsening exponent, *T* is the temperature, *E* is the activation energy for the ligament growth, *R* is the gas constant, *D*_0_ and *K* are constants. According to the formula, a linear law between ln*d* and ln*t* can be established. In addition, the linear laws were revealed between logarithm of the pore sizes and logarithm of the dealloying time [57], as well as between logarithm of the ligament width and reciprocal of the dealloying temperature [48]. Generally, the ligament/pore size increases with the dealloying time/temperature. This corollary has been verified by some studies [22,56].

There are also some indirect factors to influence the microstructure of dealloyed nanoporous metals. These indirect factors include the parting limit (for most dealloying precursors, the critical range of the noble component in an alloy is between 20 and 60 at.%), the diffusivity of a noble metal at alloy/electrolyte interfaces, the critical potential (for the electrochemical dealloying), volume shrinks and surface cracks, and so on [69]. In general, the microstructure of nanoporous metals fabricated in dealloying process is influenced by many factors, which should be controlled carefully.

## 3. Synthesis of NPC/Metal Oxides Composites

The NPC/metal oxides composites integrate NPC and other functional metal oxides together. They can be created through various methods and have potential for application in different fields. Relatively active NPC obtained by dealloying can be easily oxidized into NPC/Cu_2_O or NPC/CuO composites by annealing in air [58,70]. It was found that heat treatments toward NPC between 200 and 600 °C led to the formation of Cu oxide (CuO and Cu_2_O) layers [58]. These oxide layers with a thickness of ten to dozens of nanometers can be produced on the surface of the 3D NPC via *in situ* thermal oxidation process. For NPC with relative density of 20%~30%, the oxide layers were mainly made up of Cu_2_O after annealing at 200 °C for 30 min, while those that were composed of CuO after annealing between 400 and 600 °C for 30 min. It was reported that the oxide layer growth thicknesses in a temperature range of 100~600 °C can be estimated using the following formula [58]:

(2) $$d_{\text{oxide}}(t)=A\exp\left(\frac{-Q}{R\times T}\right)\times t^{1/2}+d_{0}$$

where *d*_oxide_(*t*) is the thickness of the formed copper oxide as a function of time, *R* is the gas constant, *T* is the temperature, *t* is time in minutes, A is the initial coefficient with values ranging from 5.518 × 10^5^ to 6.658 × 10^7^ Å·min^−1/2^, *Q* is activation energy and *d*_0_ is the initial copper oxide thickness. Thus, the thickness of Cu oxide film on the surface of NPC can be controlled by adjusting oxidation temperature and annealing time.

Ding and co-workers [71] revealed a general corrosion strategy for the straightforward fabrication of a variety of nano-structured metal oxide through a dealloying and spontaneous oxidation method. The approach mainly involves the alloying consisting of the targeted transition metals and more active metal species, and a subsequent selective leaching of active metals in proper etching liquid. During this corrosion process, the transition metal atoms left behind will undergo spontaneous oxidation at the metal/electrolyte interface to form metal oxides. By following this route, nano-structured Co_3_O_4_, Fe_3_O_4_, WO_3_, TiO_2_ and Mn_3_O_4_ with intricate structural properties have been successfully synthesized [5,71,72,73,74,75]. To develop this corrosion strategy, Zhang *et al.* [76] reported an attempt to dealloy Cu–Fe–Al ternary alloy in alkaline solutions. As a result, NPC/(Cu,Fe)_3_O_4_ composites were obtained by a direct one-step dealloying process. These composites were composed of a NPC matrix with ligament/channel sizes of 20–40 nm and octahedral (Fe,Cu)_3_O_4_ embedded particles 600–800 nm in size. The formation of these composites can be explained by the surface diffusion of Cu adatoms (to form a NPC matrix) and oxidation of the active Fe/Cu adatoms (to form metal oxides) during dealloying.

**Figure 3:**
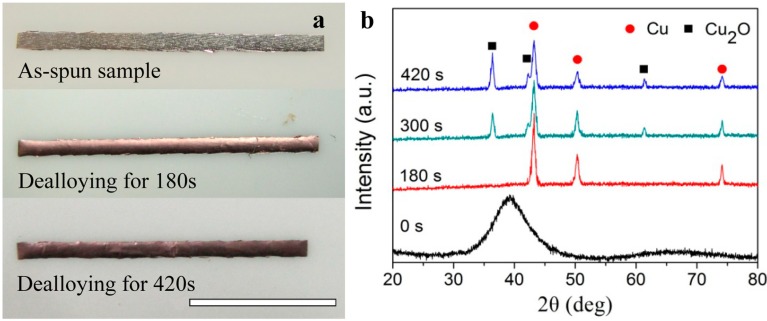
Macrophotograph (**a**) and X-ray diffraction (XRD) patterns (**b**) of the melt-spun Cu_52.5_Hf_40_Al_7.5_ ribbon before and after dealloying in oxygen-enriched 0.65 M HF for different time at 298 K open to air. Scale bar, 1 cm.

**Figure 4:**
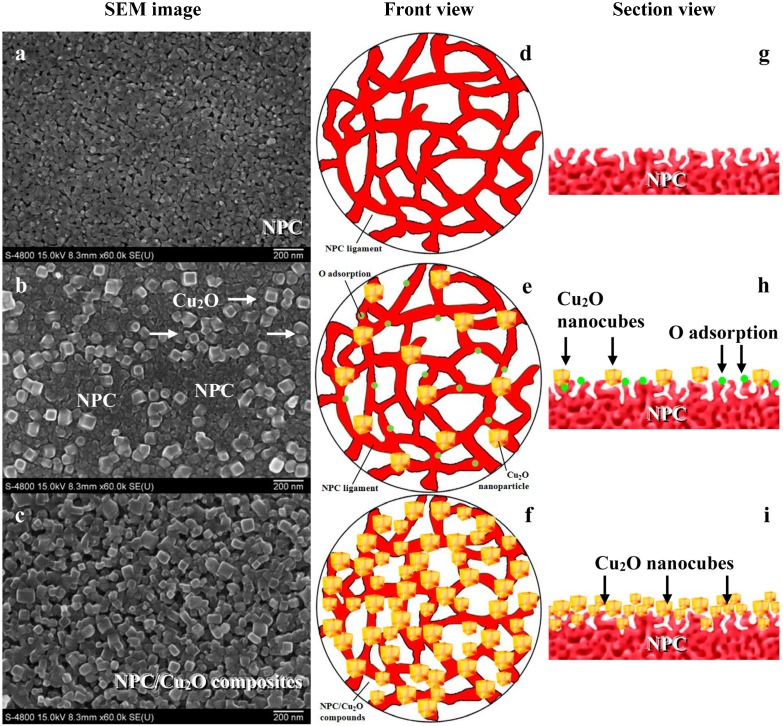
SEM (**a**–**c**) and schematic images (**d**–**i**) of Cu_52.5_Hf_40_Al_7.5_ MGs dealloying in O-enriched 0.65 M HF with different time. (**a**,**d**,**g**) 180 s; (**b**,**e**,**h**) 300 s; (**c**,**f**,**i**) 420 s.

By drawing on Ding’s corrosion strategy [71], here we report a successful fabrication of a NPC/Cu_2_O composite using the oxygen-assisted dealloying method. Unlike in our previous study [30], the HF etching solution is replenished with oxygen during the dealloying process. Then, a part of active Cu adatoms can be smoothly oxidated to Cu_2_O nanoparticles. It can be seen in Figure 3a that the color of the ribbons before and after dealloying in oxygen-enriched 0.65 M HF solutions obviously changes. With the increase of the dealloying time, the ribbon color turns from argenteous to a typical Cu metallic luster and finally changes to dark red. During the dealloying with etching time from 0 to 420 s, the ribbons keep their mechanical integrity, which is important for the subsequent applications. Figure 3b shows X-ray diffraction (XRD) patterns of Cu_52.5_Hf_40_Al_7.5_ MGs dealloying in oxygen-enriched 0.65 M HF with different etching times. The diffraction pattern of the as-spun ribbon shows a characteristic broad halo peak without appreciable crystal phase, indicating a single homogeneous amorphous structure. The SEM image (Figure 4a) of the ribbon treated in 0.65 M HF for 180 s presents nanoporous structure. The XRD pattern of the ribbon exhibits sharp crystal peaks which match with (111), (200), (220) crystal planes of Cu (JCPDS No.04-0836). However, no remaining amorphous phase is recognized in the XRD pattern. It is indicated that most of the Hf and Al elements are selectively removed from the Cu–Hf–Al precursor alloy after dealloying in 0.65 M HF for 180 s, leaving much nobler Cu element behind. As a result, the as-obtained porous metal can be identified as NPC. When the dealloying time is prolonged to 300 s, it is observed that many Cu_2_O nanocubes are formed on surfaces of the NPC (Figure 4b). When the dealloying time reaches 420 s, more Cu_2_O nanocubes coat or embed into the NPC structure to form the NPC/Cu_2_O nanocube composite (Figure 4c). These are also verified by the increasing intensity of Cu_2_O peaks in Figure 3b. Moreover, it can be seen that the dealloying product exhibits a new nanoporous composite structure.

From the above typical example, a general method to produce NPC/Cu_2_O composite structure is schematically illustrated in Figure 4d–i. The method is based on dealloying in an oxygen-enriched corrosive solution. Firstly, a 3D NPC is obtained in dealloying process. Secondly, a portion of copper on the surfaces of NPC ligaments reacts with dissolved oxygen to form Cu_2_O nanoparticles. Through the long dealloying process, a mass of Cu_2_O nanoparticles are formed on the surfaces of NPC ligaments by reaction between copper and oxygen. At last, a Cu_2_O particle layer with a certain thickness is formed on NPC surfaces. Thus, NPC/Cu_2_O composites exhibiting new nanoporous structure can be produced and can be further controlled by adjusting the dealloying time and the oxygen content in the etching solution. A similar method is also used to produce porous CuO nanoplate-films with an oxidation-assisted dealloying method [77]. It was observed that the Cu component in an alloy was oxidized preferentially into Cu_2_O nanocubes due to free oxidation by dissolved oxygen in electrolytes. Then, the Cu_2_O nanocubes are further oxidized into CuO nanoplates mainly owing to primary-cell-induced oxygen consuming corrosion. Such a method can be used universally to fabricate various porous metal oxide nanostructural films on flexible substrates for future nanostructure-based integrated circuit, sensor and solar cell applications.

**Figure 5:**
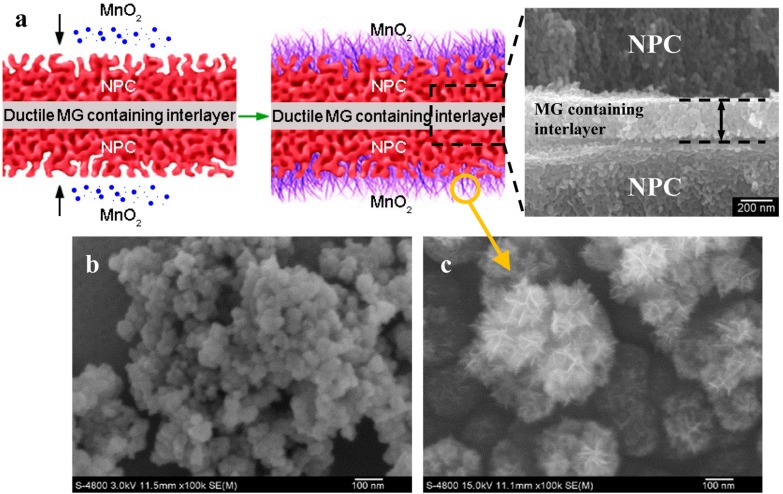
Fabrication of NPC/MnO_2_ composite [30]. (**a**) Schematic of fabrication process; (**b**) as-prepared MnO_2_ powders; (**c**) NPC/MnO_2_ composite with 50 wt.% MnO_2_.

Besides the above methods, NPC/metal oxides composites can also be synthesized through chemical deposition of metal oxides on prefabricated NPC. Recently, our group produced a new NPC-supported MnO_2_ composite (MnO_2_/NPC/MnO_2_ sandwich structure) [30]. We firstly synthesized a monolithic NPC ribbon with good mechanical integrity and bendability by designing a ductile MG-containing interlayer in the ribbon (Figure 5a). Then, the NPC was used as the substrate for the MnO_2_ deposition_._ It can be seen from Figure 5b that the as-obtained MnO_2_ prepared through the classical chemical reaction between KMnO_4_ and ethanol was composed of nanosized globular particles, which showed a serious particle aggregation phenomenon. By using a ductile NPC support, however, MnO_2_ nanoflakes (Figure 5c) were homogeneously deposited on the surface of the NPC substrate. This result indicated that the NPC with large specific surface areas and excellent electrical conductivity can effectively promote the morphological change of MnO_2_ from globular particles to nanoflakes for larger specific surface area and improve the utilization of MnO_2_ surface active sites. This method can be extended to develop more NPC/metal oxide composites with distinctive functional properties.

## 4. Synthesis of Cu_2_O Particles on Surface of MGs

So far as we know, Cu–Hf–Al MGs presented relatively good corrosion resistance in many electrolytes [78]. When dealloying Cu–Hf–Al MGs in different etching solutions, the dealloying products are diverse. As discussed in the last section, by dealloying Cu–Hf–Al MGs in 0.5 M HF solution, NPC as the general dealloying product can be fabricated. when dealloying Cu–Hf–Al MGs in HCl solutions, however, dealloying products are mainly Cu_2_O crystals with different and interesting morphologies, which are reviewed in this section. The reason why NPC cannot be produced in Cu–Hf–Al MGs by using HCl electrolytes can be explained as follows. The corrosion rates of the Cu_52.5_Hf_40_Al_7.5_ MG both in 0.5 M HCl and 0.5 M HF solutions can be estimated using the following formula [79]:

(3) $$R=\frac{87600\Delta w}{s\cdot \rho \cdot t}$$

where *R* is corrosion rate (mm·y^−1^), Δ*w* is weight loss (g), *s* is surface area of specimen (cm^2^), ρ is density of specimen (g·cm^−3^) and *t* is immersion time (h). This shows that the corrosion rate of the Cu_52.5_Hf_40_Al_7.5_ MG in 0.5 M HCl solution is about 0.2 mm·y^−1^, while it is more than 500 mm·y^−1^ in 0.5 M HF electrolyte [80]. Consequently, it is very difficult to etch Cu_52.5_Hf_40_Al_7.5_ MGs in HCl solution. On the other hand, Cu, Hf and Al elements showed noticeable potential difference in HF electrolytes, while the potential difference is small in HCl solution. For example, the potential difference between Cu and Hf in 0.5 M HF electrolytes was distinct and more than 0.8 V, which provides an advantageous driving force for the dissolution of less noble elements Hf under free corrosion condition. The potential difference between Cu and Hf in 0.5 M HCl solution, however, was less than 0.2 V [80]. Thus, it is difficult to remove Hf in HCl solution under free etching conditions. Considering the above two aspects, NPC cannot be easily synthesized by dealloying of Cu–Hf–Al MGs in HCl electrolytes.

### 4.1. Synthesis of Regular Cu_2_O Particles on Surface of MGs

Xue *et al.* [81] found that Cu_2_O, the initially formed product of Cu oxidation in air or oxygen atmosphere, can spontaneously grow at the surface of Cu foil in Cl^−^ solutions at room temperature. By adjusting concentrations of Cl^−^ ions, Cu_2_O crystals with various shapes including octahedra, rhombic dodecahedra and spheres are produced and can be tailored on the surface of Cu foil. Using the salutary experience of this route, we found that various and regular Cu_2_O particles can also be fabricated on surfaces of MGs by dealloying Cu-based MG ribbons in the HCl solution with low concentrations for different times. For example, by dealloying Cu_52.5_Hf_40_Al_7.5_ MG ribbons in 0.05 M diluted HCl solution for 4 h, 5 h, 6 h, 8 h, 14 h, 20 h and 24 h, Cu_2_O crystals with truncated tetrahedron, cube, cuboctahedron, truncated octahedron, octahedron, hexapod and octahedron-detached hexapod shapes [32,33] were synthesized, respectively. So, regular Cu_2_O particles with designable morphology can be tailored in the diluted HCl solution by simply controlling dealloying time.

The characteristics of these regular Cu_2_O crystals are listed in Table 2. It can be seen that the 3D sizes of Cu_2_O crystals do not change much in the first 8 h of dealloying though they show different edge lengths. Then, the sizes of Cu_2_O crystals increase obviously after dealloying for 14 h, and get bigger with the increase in dealloying time. It should be noted that the volume fraction of Cu_2_O particles on MG surface is low (less than 20%) and does not change much with the extension of etching time. Since the Cu_2_O crystals with different morphologies possess important electrical and optical properties [82,83], it is necessary to enhance the volume fraction of Cu_2_O particles on the glassy surface.

**Table 2:** Characteristics of regular Cu_2_O crystals produced by free dealloying of Cu_52.5_Hf_40_Al_7.5_ MG in 0.05 M HCl solution for different times at 298 K open to air [32,33].

**Dealloying Time/h**	4	5	6	8	14	20	24
**Morphology**	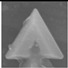	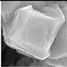	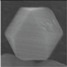	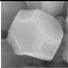	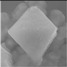	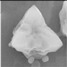	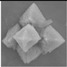
	Truncated tetrahedron	Cube	Cuboctahedron	Truncated octahedron	Octahedron	Hexapods	Octahedron-detached hexapods
**Edge length/nm**	~300	~300	~300	~150	~450	~500	~1100
**Volume fraction/%**	10.6	13.3	12.2	13.9	15.8	14.4	19.8 (mixed with other shapes)

### 4.2. Preparation of Cu_2_O Micro-flowers on Surface of MGs

In order to improve the volume fraction of Cu_2_O particles on glassy surfaces, Cu-based MG ribbons dealloying in HCl solutions with enhanced concentration were studied [34]. After Cu_52.5_Hf_40_Al_7.5_ glassy ribbons were dealloyed in 0.1 M, 0.2 M and 0.4 M HCl solution for 8 h, Cu_2_O particles formed on MG surfaces exhibited a flower-like shape. The mean surface coverage rate of Cu_2_O micro-flowers increased from 17.2% to 33.1% with the increase in HCl concentration. The Cu_2_O coverage rate was improved compare with the above work (13.9%, 0.05 M HCl for 8 h) [32]. The improvement in the Cu_2_O coverage rate arose from the increased HCl concentration that promoted the dealloying reaction including the reaction speed and reaction product. As a result, the sizes of Cu_2_O crystals gradually increased and Cu_2_O crystals in regular polyhedral shapes could not be maintained but rather grow into micro-flowers. On the other hand, Cu_52.5_Hf_40_Al_7.5_ glassy ribbons dealloying in 0.5 M HCl solution for 8 h, 14 h and 20 h were also studied [34]. When the dealloying time was extended from 8 h to 14 h, some cracks were formed on the glassy surface. Furthermore, deeper cracks were observed in the ribbon surface dealloyed for 20 h. It was found that plentiful Cu_2_O/CuO particles grew up from these crack walls. With the increase of the dealloying time, the mean surface coverage rate of Cu*_x_*O (*x* = 1,2) crystals increased gradually.

**Figure 6:**
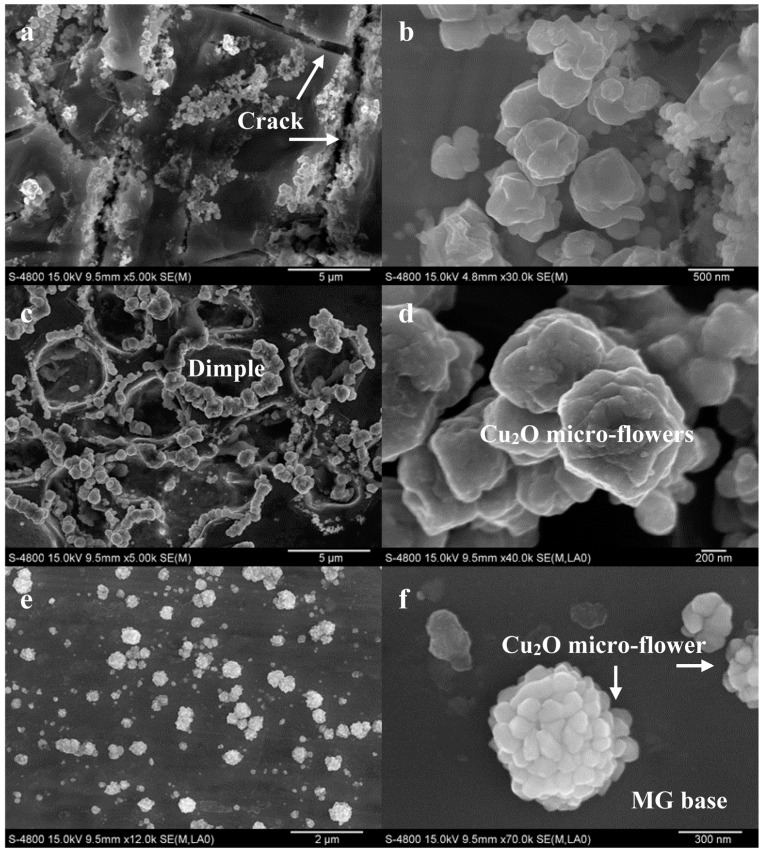
SEM images of Cu-based MG dealloying in 1.2 M HCl solution at 298 K. (**a**,**b**) Cu_52.5_Hf_40_Al_7.__5_ MG, dealloying for 1.5 h; (**c**,**d**) Cu_52.5_Hf_40_Al_5_Nb_2.5_ MG, dealloying for 14 h; (**e**,**f**) Cu_50_Hf_40_Al_5_Nb_5_ MG, dealloying for 14 h.

From the above results, we can conclude that higher concentration of HCl solutions and longer dealloying time are beneficial for improving the volume fraction of Cu_2_O particles on glassy surfaces. However, the cracks formed on the Cu–Hf–Al glassy surfaces in concentrated HCl solutions are unavoidable. They are harmful to the mechanical integrity of an alloy ribbon. To solve this problem, dealloying attempts to Cu–Hf–Al–Nb glassy ribbons are made. For Cu_52.5_Hf_40_Al_7.__5_ glassy ribbon dealloying in 1.2 M HCl for only 1.5 h (Figure 6a,b), many cracks form on the ribbon surface. Cu_2_O micro-flowers with diameter about 600 nm grow up from the crack walls. For Cu_52.5_Hf_40_Al_5_Nb_2.5_ glassy ribbon dealloying in 1.2 M HCl for 14 h (Figure 6c,d), dimples instead of cracks are present on the ribbon surface. Cu_2_O micro-flowers with the biggest size to 1.1 μm grow along the edges of the dimples. For Cu_50_Hf_40_Al_5_Nb_5_ glassy ribbon dealloying in 1.2 M HCl for 14 h (Figure 6e,f), however, a smooth surface is retained. Cu_2_O micro-flowers with diameter less than 700 nm are created on the glassy surface. The volume fraction of Cu_2_O crystals on glassy surfaces reaches 39% ± 5 %. Thus, a composite with an ideal glassy surface plus a relatively high volume fraction of Cu_2_O micro-flowers is successfully synthesized.

**Figure 7:**
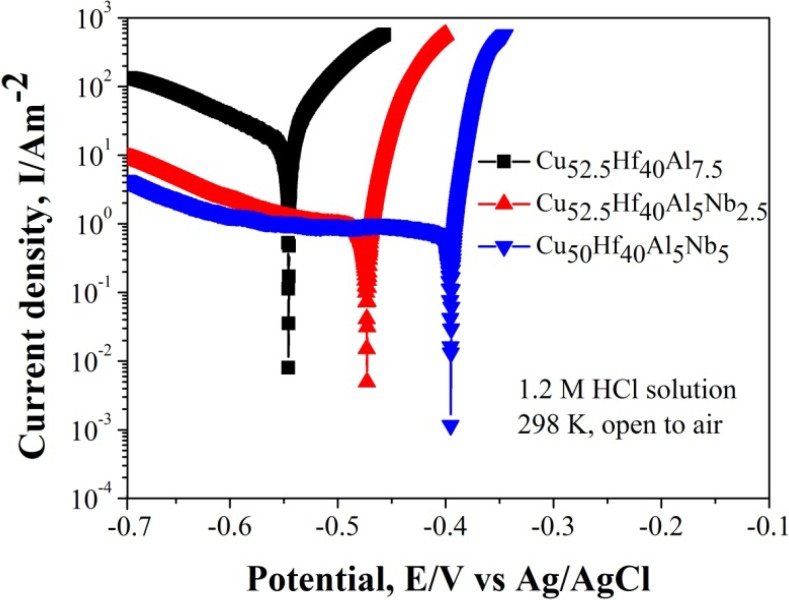
Potentiodynamic polarization curves of Cu–Hf–Al(–Nb) MG in 1.2 M HCl solution at 298 K open to air.

Figure 7 shows potentiodynamic polarization curves of Cu–Hf–Al(–Nb) glassy ribbons in 1.2 M HCl solution. With the increase of the Nb content, MG samples present lower corrosion current density and more positive corrosion potential, indicating a big improvement in the corrosion resistance of the MG samples. So, Cu_50_Hf_40_Al_5_Nb_5_ glassy ribbon with good corrosion resistance in 1.2 M HCl solution can maintain its smooth surface during the long corrosion process. To our knowledge, MG is a good carrier for Cu_2_O particles, because MG exhibits high strength, high toughness and high corrosion resistance. In addition, the Cu_2_O crystals formed on MG surfaces are easier to store or extract as compared to traditional chemical methods. Consequently, the MG/Cu_2_O micro-flower compounds contain multiple potential properties which are well worth developing.

Dealloying products of Cu-based MG ribbons in HF and HCl solutions are summarized in Table 3. By choosing different Cu-based MGs and etching conditions, various dealloying products with different surface and inner compositions can be tailored. These dealloying products with multiple functionalities could be applied in wide fields in near future.

**Table 3:** Summary of dealloying products of Cu-based MG in acidic solutions at 298 K.

Etching Solution	Dealloying Products		MG	Dealloying Conditions	References
	Inner	Surface			
HF	NPC	NPC	Cu_52.5_Hf_40_Al_7.5_	0.5 M HF, 300 s	[30]
	NPC	Regular Cu_2_O particles	Cu_52.5_Hf_40_Al_7.5_	oxygen-enriched 0.65 M HF, 420 s	This study
HCl	MG	Regular Cu_2_O particles	Cu_52.5_Hf_40_Al_7.5_	0.05 M HCl, 4~24 h	[32,33]
	MG	Cu_2_O micro-flowers	Cu_50_Hf_40_Al_5_Nb_5_	1.2 M HCl, 14 h	This study

## 5. Applications of Dealloying Products

The paper reviews representative dealloying products of Cu-based MGs in acidic solutions, such as NPC, NPC/Cu_2_O composites and metallic glass-supported Cu_2_O composites with tunable Cu_2_O shapes. These new dealloying products with unique structures and multiple properties would provide a wide array of possible applications in many areas.

Nanoporous metals, as the most famous dealloying products, have been the focus of much attention due to their potential in various applications. So far, the study on nanoporous gold (NPG) makes up a high proportion of dealloying works. Just in recent years, fabrication and application development of inexpensive NPC have attracted considerable attention from many researchers. Representative applications of NPC and composites are listed in Table 4. The first significant development toward NPC was reported by Chen’s group [22]. It was found that the tunable nanoporosity leads to a remarkable improvement in surface-enhanced Raman scattering (SERS) of NPC, which was helpful in developing inexpensive SERS substrates for sensitive instrumentations in molecular diagnostics. After that, diversified reports about NPC applications were unveiled. For example, NPC was considered to be a good support for horseradish peroxidase immobilization [84]. Moreover, NPC-contained composites presented superior catalytic and sensitive performance in oxidation of hydrazine and alcohols, degrading organic compounds and detecting glucose [50,85,86,87,88].

**Table 4:** Representative applications of NPC and composites.

Products	Application Field	References
NPC	Surface-enhanced Raman scattering (SERS)	[22,89]
	Immobilization of horseradish peroxidase	[84]
NPC/Ag core–shell composite	SERS	[90]
NPC/Pt(Pd) core–shell composite	Methanol electro-oxidation	[91]
NPC/Au core-shell composite	Electrocatalysis and nonenzymatic biosensing	[87]
Ni-B amorphous nanoparticles modified NPC	Ethanol oxidation	[88,92]
NPC/Cu composite	Electro-oxidation of hydrazine	[85]
Nanoporous Cu–O system	Catalysts towards CO oxidation	[93]
NPC/Cu_2_O nanocomposite	Adsorption of methyl orange	[94]
NPC/MG composite	Degradation of azo dye	[86]
	Degradation of phenol	[50]
NPC/Si composite	Lithium-ion battery anodes	[95]
NPC/Cu_2_O composite		[70]
Cu/NPC/MnO_2_ composite		[96]
NPC/MnO_2_ composite	Supercapacitor electrodes	[30]
NPC/(Fe,Cu)_3_O_4_ composites	Excellent magnetic/electrical properties for potential applications in sensors, information storage, and so on	[76]

Besides these developments, an important application field for NPC is energy storage, especially for electrochemical supercapacitors (ECs). As charge-storage devices, ECs possess a unique combination of high power, high energy and long lifetimes [97,98]. They were widely used in portable electronics and hybrid electric vehicles [99,100]. There are two basic kinds of ECs: one is double-layer capacitance relying on surface ion adsorption, such as carbon based materials (e.g., carbon nanotubes, graphene, porous carbon, carbon aerogel and active carbon fiber) [101,102,103,104,105]; another is pseudo-capacitance relying on surface redox reactions, including metal oxides (e.g., RuO_2_, MnO_2_, Co_3_O_4_ and NiO) [106,107,108,109] and conducting polymers (e.g., polyaniline, polypyrrole and polythiophene) [110,111,112]. It should be noted that the capacitive performances of ECs depend on the effective surface area and conductivity of electrode materials [98]. Thus, NPG with a high specific surface area and high conductivity has been firstly tried as a current collector or a substrate support for the electrode materials in supercapacitors. It was reported that bare NPG-based double-layer supercapacitors had excellent charge-discharge cycling stability, although their capacitance was still too low (~5 F·g^−1^) for real applications [113,114]. For enhancing capacitive performance, pseudo-capacitive materials with high theoretical values such as MnO_2_, polyaniline and polypyrrole (PPy) were deposited on the high-surface-area and highly conductive NPG surface [115,116,117]. These nanocomposite-based supercapacitors exhibit high capacitance properties (e.g., a specific capacitance of ~1145 F·g^−1^ for NPG/MnO_2_, a power density of 296 kW·kg^−1^ for NPG/PPy). However, Au is a well-known noble metal, which would limit its large-scale application. With the aim of reducing the cost and promoting the practical application of nanoporous metals in the EC field, nanoporous Ni has been fabricated by dealloying Mn–Ni, Al–Ni, Cu–Ni, Fe–Ni, Mg–Ni, Zn–Ni and Mn–Ni–Cu alloy in different solutions [118,119,120,121]. The nanoporous Ni as an electrode substrate presented a stable areal capacitance (1.7 F·cm^−2^) [122]; however, a very small pore size (usually less than 10 nm) inhibited the growth of the pseudo-capacitive materials inside the nanopores. Therefore, it is expected that the inexpensive nanoporous Cu (NPC) with tunable pore size in a wide range from 10 to 60 nm could be considered as an excellent substrate support for pseudo-capacitive materials.

**Figure 8:**
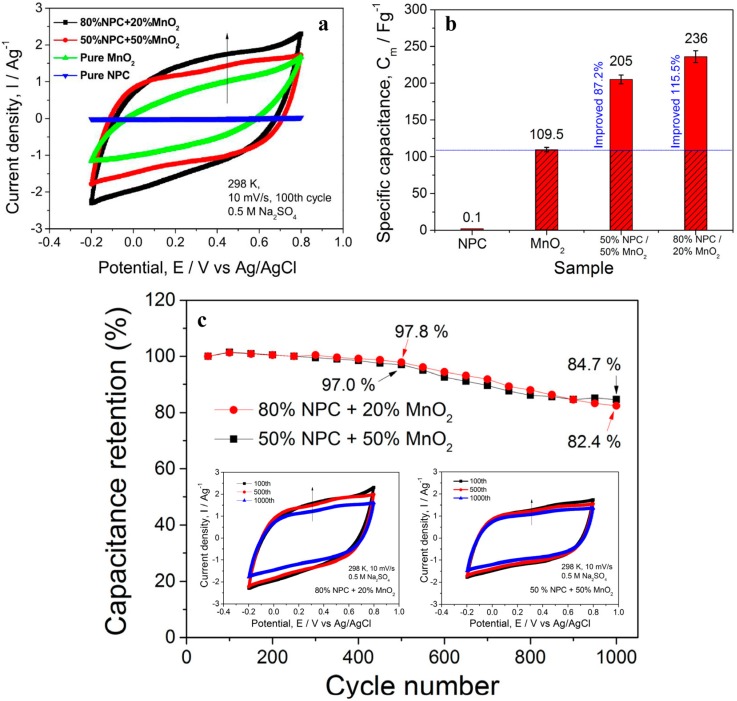
NPC/MnO_2_ composite for ECs. (**a**) CV curves of monolithic NPC, pure MnO_2_ and NPC/MnO_2_ composites with different weight ratios in 0.5 M Na_2_SO_4_ solution; (**b**) specific capacitance of monolithic NPC, pure MnO_2_ and NPC/MnO_2_ composites with different weight ratios; (**c**) cycling performance of the NPC/MnO_2_ composite at the scan rate of 10 mV·s^−1^; the inserts show CV curves of NPC/MnO_2_ composites with different weight ratios in 0.5 M Na_2_SO_4_ solution at different scan cycles. Adapted with permission from Elsevier, Copyright 2015 [30].

In our recent work, a new NPC-supported MnO_2_ (NPC/MnO_2_) composite for ECs was produced [30]. It can be clearly seen from the cyclic voltammogram (CV) curves (Figure 8a) that the capacitive current of the NPC in the voltage window was negligibly low, indicating NPC was only used as a stable substrate. On the other hand, it was surprisingly found that the closed area of CV curves (Figure 8a) and the specific capacitance values (Figure 8b) of the NPC/MnO_2_ composite were remarkably enhanced as compared to that of the pure MnO_2_ powders. When combining the electrochemical performance of pure MnO_2_ and NPC/MnO_2_ composites with their corresponding surface morphologies and distribution of MnO_2_ (Figure 5b,c), it was indicated that NPC substrate can efficiently improve the utilization of MnO_2_ surface active sites and promote MnO_2_ chemical reactions. The cycling stability of NPC/MnO_2_ composites with different weight ratios is presented in Figure 8c. The specific capacitance for the two composites remained greater than 97% and 82% of the initial value after 500 and 1000 cycles, respectively. All the results showed that the as-obtained NPC/MnO_2_ composite had a commendable potential for EC application. In future works, the optimization of the coated capacitive materials, including their amount and structure, could further enhance their capacitive performance and accelerate these inexpensive ECs for a practical application.

In NPC/MnO_2_ composite supercapacitor electrodes, the NPC, as a current collector, was the support for MnO_2_ nanoparticles. The presence of NPC changed the morphology of MnO_2_ and improved the utilization of surface active sites of MnO_2_. This is of great significance to develop electrode materials with high electrochemical performance. On the other hand, it is known that NPC is unstable and has a tendency to form Cu_2_O and CuO under high voltage. Using this, Cu/Cu_2_O composites [123,124] and Cu/CuO composites [125] were successfully developed for pseudocapacitance electrodes. The pseudocapacitance effect of these composites came from the redox transformation between the Cu^+^ and Cu^2+^ species in the process of charging and discharging. As a result, the synergistic effect of Cu oxide and MnO_2_ contributed to the pseudocapacitance properties of the composites, showing a low cost method for improvement of pseudocapacitance properties. Moreover, in comparison with carbon-based supercapacitor, the NPC-based supercapacitor exhibits distinct merits. Although carbon is a kind of low cost material and exhibits ultra-high specific capacitance, the production of carbon nanotubes, graphene, *etc.* includes a complex preparation process, expensive equipment, and low yield. Thus, it is difficult to achieve mass production of carbon-based supercapacitors at low cost. In contrast, the preparation of NPC is based on free dealloying, which is a very simple and rapid fabrication method. That makes NPC-based supercapacitors economical and practical. As a result, the development of NPC-based supercapacitors with low cost, stable properties and long lifetimes is of great importance.

An NPC used in lithium-ion batteries is another energy storage material. The fabrication of nanoporous metal-based composites for lithium-ion batteries is still in its early stages. Lang’s team [96] uncovered a flexible Cu/NPC/MnO_2_ hybrid bulk electrode for high-performance lithium-ion batteries, which showed a capacity as high as ~1100 mA·h·g^−1^ for 1000 cycles. In addition, it was reported that an NPC supported cuprous oxide prepared by the direct oxidation of NPC at high temperatures was applied in high-performance lithium ion battery anodes [70].

Up to now, the majority of research about applications of Cu-containing alloys for dealloying products has focused on NPC and NPC-based composites. Application development in broader fields for other dealloying products, such as NPC/Cu_2_O composites and MG/Cu_2_O composites with tailored Cu_2_O shapes, needs to be carried out in the near future. The corresponding experimental results will be presented soon by our group.

## 6. Conclusions and Outlook

**Figure 9:**
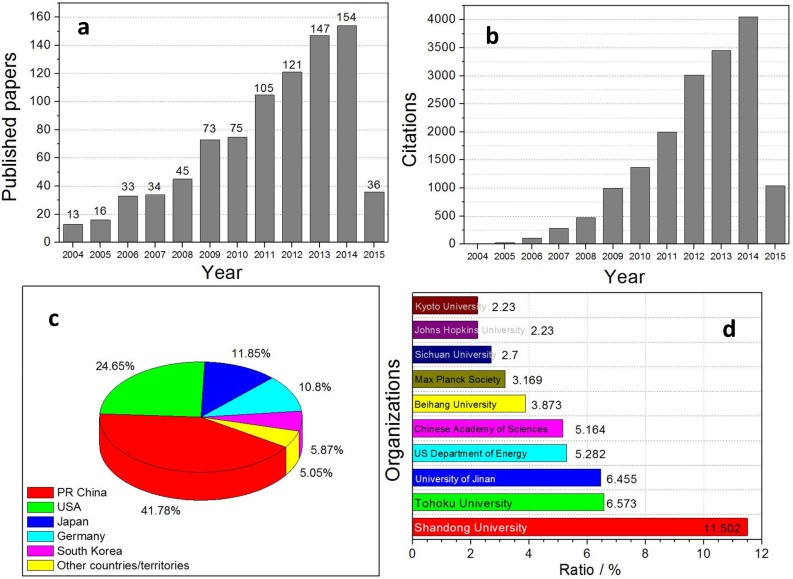
Analyses of publications in the dealloying field using the topic of “dealloying” (1 January 2004–31 March 2015). (**a**) Papers published in each year; (**b**) citations in each year; (**c**) pie chart showing the paper publication ratio of different countries/territories; (**d**) bar graph showing the paper publication ratio of different organizations.

The fabrication and applications of nanoporous metals and composites with dealloying techniques have earned more and more attention from academic research. These dealloying products show good performance in many fields. Although the history of research into dealloying is very short, the achievements gained in such a short period of time are amazing. According to the data from the “Web of Science” website (Figure 9), there have been 852 papers published in the dealloying field in the last 10 years (1 January 2004–31 March 2015). Every paper is cited nearly 20 times. In addition, the “H-index” is 63, which is far above average. The number of published papers and citations has risen over the last several years. In 2014, there were more than 150 published papers and over 4000 total citations. All the statistics show dealloying studies to be a hot topic in academia. As for papers published in countries/territories, the number of papers related to dealloying published by China, the USA, Japan and Germany is significantly higher than those of other countries/territories. In particular, papers in China account for more than forty percent. Per organization, Shandong University and Tohoku University, as leaders in the study of dealloying, have published the most papers in the world. The popularity of study in the dealloying field is thanks to rapid developments in the fields of energy, catalysis, biosensing, *etc.* Dealloying has become a multidisciplinary and interdisciplinary research. Dealloying research is also attracting more and more researchers from different disciplines. With sufficient cooperation, there will be more surprises in the near future.

In this paper, the dealloying products via free-dealloying of Cu-based metallic glasses in acid solutions are summarized. With different dealloying conditions, various dealloying products including NPC, NPC/Cu_2_O composites and MG/Cu_2_O composites are fabricated. NPC with uniform pore structure can be obtained by dealloying because of homogeneous structure of MG precursors. The ligament/pore size of dealloyed NPC can be tailored by the addition of different trace elements into MGs. A noble element such as Ag additive in MGs results in the formation of a nanoprous Cu–Ag alloy with ultrafine porous structure. An active element such as Al additive, however, leads to the formation of a wider ligament of NPC. Besides alloy compositions, many factors, such as dealloying solutions, etching time, dealloying temperatures and so on, have significant influence on the ligament/pore size of the resulting NPC. The change in ligament/pore size will influence the performance of the NPC in many application fields. Consequently, the application properties of NPC can be tailored by simply changing these factors. In addition, when dealloying in an oxygen-enrichment solution, metal oxide can be formed. In this study, NPC/Cu_2_O composites are synthesized by this method, which is called the dealloying and spontaneous oxidation method. The electrochemical properties of the composites need to be further developed. Meanwhile, MG presents excellent corrosion resistance performance in an appropriate corrosion environment. In this situation, MG ribbon with good mechanical integrity can be retained after corrosion, which provides an opportunity for the formation of MG-based compounds. The application fields of these compounds deserve to be further studied.

Energy storage is one of the most important and meaningful application fields of dealloying products. NPC possesses excellent electrical conductivity, and can be used as a current collector and support material for metal oxide. Thus, the as-obtained NPC/metal oxide composite material can be used as a low-cost supercapacitor and lithium ion battery electrodes. Through the combination of dealloying with other pore-forming technology, nano/mirco-porous Cu or so called bimodal porous Cu can be prepared. This type of composite with porous structure is expected to have a more outstanding performance in the field of energy storage. In lithium-air batteries, for example, the big pores can accommodate more discharge product (Li_2_O_2_) to enhance the energy density, while the small ligament/pore with high catalytic activity can effectively catalyze the reduction of Li_2_O_2_ into Li during the charge process to enhance the cycling stability [98]. At the same time, the porous structured composite can be used as a substrate to form nano/micro-scaled metal oxides with different morphologies using diverse techniques, such as anodization, heat treatment, chemical deposition and so on. The series of compounds are expected to show improved performance in energy storage.

## References

[1] Zhang C., Sun J., Xu J., Wang X., Ji H., Zhao C., Zhang Z. (2012). Formation and microstructure of nanoporous silver by dealloying rapidly solidified Zn–Ag alloys. Electrochim. Acta.

[2] Darque-Ceretti E., Felder E., Aucouturier M. (2011). Foil and leaf gilding on cultural artifacts: Forming and adhesion. Matéria.

[3] Ding Y., Kim Y., Erlebacher J. (2004). Nanoporous gold leaf: “Ancient technology”/advanced material. Adv. Mater..

[4] Xu C., Liu Y., Wang J., Geng H., Qiu H. (2012). Nanoporous PdCu alloy for formic acid electro-oxidation. J. Power Sources.

[5] Xu C., Liu Y., Zhou C., Wang L., Geng H., Ding Y. (2011). An *in situ* dealloying and oxidation route to Co_3_O_4_ nanosheets and their ambient-temperature CO oxidation activity. ChemCatChem.

[6] Lang X., Fu H., Hou C., Han G., Yang P., Liu Y., Jiang Q. (2013). Nanoporous gold supported cobalt oxide microelectrodes as high-performance electrochemical biosensors. Nat. Commun..

[7] Pickering H., Wagner C. (1967). Electrolytic dissolution of binary alloys containing a noble metal. J. Electrochem. Soc..

[8] Seker E., Reed M., Begley M. (2009). Nanoporous gold: Fabrication, characterization, and applications. Materials.

[9] Hakamada M., Nakano H., Furukawa T., Takahashi M., Mabuchi M. (2010). Hydrogen storage properties of nanoporous palladium fabricated by dealloying. J. Phys. Chem. C.

[10] Jin H., Kramer D., Ivanisenko Y., Weissmüller J. (2007). Macroscopically strong nanoporous Pt prepared by dealloying. Adv. Eng. Mater..

[11] Li Z., Wang X., Lu X. (2014). Refinement of nanoporous silver by adding surfactant to the electrolyte. ECS Electrochem. Lett..

[12] Dan Z., Qin F., Yamaura S., Xie G., Makino A., Hara N. (2014). Refinement of nanoporous copper by dealloying MgCuY amorphous alloys in sulfuric acids containing polyvinylpyrrolidone. J. Electrochem. Soc..

[13] Gu X., Xu L., Tian F., Ding Y. (2009). Au–Ag alloy nanoporous nanotubes. Nano Res..

[14] Koczkur K., Yi Q., Chen A. (2007). Nanoporous Pt–Ru networks and their electrocatalytical properties. Adv. Mater..

[15] Xu C., Liu Y., Su F., Liu A., Qiu H. (2011). Nanoporous PtAg and PtCu alloys with hollow ligaments for enhanced electrocatalysis and glucose biosensing. Biosens. Bioelectron..

[16] Zhang Z., Wang Y., Wang X. (2011). Nanoporous bimetallic Pt–Au alloy nanocomposites with superior catalytic activity towards electro-oxidation of methanol and formic acid. Nanoscale.

[17] Chen X., Jiang Y., Sun J., Jin C., Zhang Z. (2014). Highly active nanoporous Pt-based alloy as anode and cathode catalyst for direct methanol fuel cells. J. Power Sources.

[18] Tang Y., Tang B., Qing J., Li Q., Lu L. (2012). Nanoporous metallic surface: Facile fabrication and enhancement of boiling heat transfer. Appl. Surf. Sci..

[19] Biener J., Wittstock A., Zepeda-Ruiz L.A., Biener M.M., Zielasek V., Kramer D., Viswanath R.N., Weissmüller J., Bäumer M., Hamza A.V. (2009). Surface-chemistry-driven actuation in nanoporous gold. Nat. Mater..

[20] Kang J., Hirata A., Qiu H., Chen L., Ge X., Fujita T., Chen M. (2014). Self-grown oxy-hydroxide@nanoporous metal electrode for high-performance supercapacitors. Adv. Mater..

[21] Chen L., Fujita T., Chen M. (2012). Biofunctionalized nanoporous gold for electrochemical biosensors. Electrochim. Acta.

[22] Chen L., Yu J., Fujita T., Chen M. (2009). Nanoporous copper with tunable nanoporosity for SERS applications. Adv. Funct. Mater..

[23] Erlebacher J., Aziz M., Karma A., Dimitrov N., Sieradzki K. (2001). Evolution of nanoporosity in dealloying. Nature.

[24] Pia G., Delogu F. (2015). Nanoporous Au: Statistical analysis of morphological features and evaluation of their influence on the elastic deformation behavior by phenomenological modeling. Acta Mater..

[25] Detsi E., Punzhin S., Rao J., Onck P., Hosson J. (2012). Enhanced strain in functional nanoporous gold with a dual microscopic length scale structure. ACS Nano.

[26] Detsi E., Chen Z., Vellinga W., Onck P., Hosson J. (2011). Reversible strain by physisorption in nanoporous gold. Appl. Phys. Lett..

[27] Biener J., Hodge A., Hayes J., Volkert C., Zepeda-Ruiz L., Hamza A., Abraham F. (2006). Size effects on the mechanical behavior of nanoporous Au. Nano Lett..

[28] Kong Q., Lian L., Liu Y., Zhang J. (2014). Fabrication and compression properties of bulk hierarchical nanoporous copper with fine ligament. Mater. Lett..

[29] Weissmüller J., Wang K. (2013). Composites of nanoporous gold and polymer. Adv. Mater..

[30] Wang Z., Liu J., Qin C., Liu L., Zhao W., Inoue A. (2015). Fabrication and new electrochemical properties of nanoporous Cu by dealloying amorphous Cu–Hf–Al alloys. Intermetallics.

[31] Qin C., Wang Z., Liu H., Liu L., Wang H., Ding J., Zhao W. (2014). Monolithic nanoporous copper with novel electrochemical properties fabricated by dealloying Cu–Zr(–Al) metallic glasses. Mater. Sci. Forum.

[32] Wang Z., Qin C., Zhao W., Jia J. (2012). Tunable Cu_2_O nanocrystals fabricated by free dealloying of amorphous ribbons. J. Nanomater..

[33] Wang Z., Wang L., Qin C., Liu J., Li Y., Zhao W. (2014). Tailored dealloying products of Cu-based metallic glasses in hydrochloric acid solutions. Mater. Res..

[34] Wang Z., Qin C., Liu L., Wang L., Ding J., Zhao W. (2014). Synthesis of Cu*_x_*O (*x* = 1,2)/amorphous compounds by dealloying and spontaneous oxidation method. Mater. Res..

[35] Li M., Zhou Y.Z., Geng H. (2012). Fabrication of nanoporous copper ribbons by dealloying of Al–Cu alloys. J. Porous Mater..

[36] Liu W., Zhang S., Li N., Zheng J., An S., Li G. (2012). Influence of dealloying solution on the microstructure of monolithic nanoporous copper through chemical dealloying of Al 30 at.% Cu alloy. Int. J. Electrochem. Sci..

[37] Tan X., Li K., Niu G., Yi Z., Luo J., Liu Y., Han S., Wu W., Tang Y. (2012). Effect of heat treatment of Mn–Cu precursors on morphology of dealloyed nanoporous copper. J. Cent. South Univ..

[38] Rizzi P., Scaglione F., Battezzati L. (2014). Nanoporous gold by dealloying of an amorphous precursor. J. Alloy. Compd..

[39] Scaglione F., Rizzi P., Celegato F., Battezzati L. (2014). Synthesis of nanoporous gold by free corrosion of an amorphous precursor. J. Alloy. Compd..

[40] Qiu H., Peng L., Li X., Xu H., Wang Y. (2015). Using corrosion to fabricate various nanoporous metal structures. Corros. Sci..

[41] Feng Y., Zhang S., Xing Y., Liu W. (2012). Preparation and characterization of nanoporous Cu_6_Sn_5_/Cu composite by chemical dealloying of Al–Cu–Sn ternary alloy. J. Mater. Sci..

[42] Liu W., Zhang S., Li N., Zheng J., An S., Xing Y. (2012). Formation of nanoporous copper with hybrid-modal pore size distributions related to surface diffusion of copper atoms during dealloying of Mg 13.5 at.% Cu alloy in an acidic solution. Int. J. Electrochem. Sci..

[43] Hayes J., Hodge A., Biener J., Hamza A., Sieradzki K. (2006). Monolithic nanoporous copper by dealloying Mn–Cu. J. Mater. Res..

[44] Qi Z., Zhao C., Wang X., Lin J., Shao W., Zhang Z., Bian X. (2009). Formation and characterization of monolithic nanoporous copper by chemical dealloying of Al–Cu alloys. J. Phys. Chem. C.

[45] Zhao C., Qi Z., Wang X., Zhang Z. (2009). Fabrication and characterization of monolithic nanoporous copper through chemical dealloying of Mg–Cu alloys. Corros. Sci..

[46] Lin B., Kong L., Hodgson P., Dumée L. (2014). Impact of the de-alloying kinetics and alloy microstructure on the final morphology of de-alloyed meso-porous metal films. Nanomaterials.

[47] Dan Z., Qin F., Sugawara Y., Muto I., Hara N. (2012). Fabrication of nanoporous copper by dealloying amorphous binary Ti–Cu alloys in hydrofluoric acid solutions. Intermetallics.

[48] Dan Z., Qin F., Sugawara Y., Muto I., Hara N. (2014). Dependency of the formation of Au-stabilized nanoporous copper on the dealloying temperature. Microporous Mesoporous Mater..

[49] Luo X., Li R., Liu Z., Huang L., Shi M., Xu T., Zhang T. (2012). Three-dimensional nanoporous copper with high surface area by dealloying Mg–Cu–Y metallic glasses. Mater. Lett..

[50] Deng Z., Zhang C., Liu L. (2014). Chemically dealloyed MgCuGd metallic glass with enhanced catalytic activity in degradation of phenol. Intermetallics.

[51] Abe H., Sato K., Nishikawa H., Takemoto T., Fukuhara M., Inoue A. (2009). Dealloying of Cu–Zr–Ti bulk metallic glass in hydrofluoric acid solution. Mater. Trans..

[52] Aburada T., Fitz-Gerald J., Scully J. (2011). Synthesis of nanoporous copper by dealloying of Al–Cu–Mg amorphous alloys in acidic solution: The effect of nickel. Corros. Sci..

[53] Dan Z., Qin F., Makino A., Sugawara Y., Muto I., Hara N. (2014). Fabrication of nanoporous copper by dealloying of amorphous Ti–Cu–Ag alloys. J. Alloy. Compd..

[54] Zhang Q., Zhang Z. (2010). On the electrochemical dealloying of Al-based alloys in a NaCl aqueous solution. Phys. Chem. Chem. Phys..

[55] Wang Y., Zhang W., Inoue A. (2012). Nanoporous Cu wide ribbons with good mechanical integrity. Mater. Sci. Eng. B.

[56] Luo X., Li R., Huang L., Zhang T. (2013). Nucleation and growth of nanoporous copper ligaments during electrochemical dealloying of Mg-based metallic glasses. Corros. Sci..

[57] Dan Z., Qin F., Sugawara Y., Muto I., Makino A., Hara N. (2013). Nickel-stabilized nanoporous copper fabricated from ternary TiCuNi amorphous alloys. Mater. Lett..

[58] Cheng I., Hodge A. (2012). Morphology, oxidation, and mechanical behavior of nanoporous Cu foams. Adv. Eng. Mater..

[59] Zhang X., Li Y., Liu Y., Zhang H. (2013). Fabrication of a bimodal micro/nanoporous metal by the Gasar and dealloying processes. Mater. Lett..

[60] Liu W., Zhang S., Li N., Zheng J., Xing Y. (2011). Influence of phase constituent and proportion in initial Al–Cu alloys on formation of monolithic nanoporous copper through chemical dealloying in an alkaline solution. Corros. Sci..

[61] Zhao C., Wang X., Qi Z., Ji H., Zhang Z. (2010). On the electrochemical dealloying of Mg–Cu alloys in a NaCl aqueous solution. Corros. Sci..

[62] Aburada T., Unlu N., Fitz-Gerald J., Scully J. (2008). Effect of Ni as a minority alloying element on the corrosion behavior in Al–Cu–Mg–(Ni) metallic glasses. Scr. Mater..

[63] Dan Z., Qin F., Sugawara Y., Muto I., Hara N. (2014). Uniform evolution of nanoporosity on amorphous Ti–Cu alloys. J. Nanosci. Nanotechnol..

[64] Lan G., Xie Z., Huang Z., Yang S., Zhang X., Zeng Y., Jiang J. (2014). Amorphous alloy: Promising precursor to form nanoflowerpot. Adv. Mater. Sci. Eng..

[65] Dan Z., Qin F., Hara N. (2014). Refinement of nanoporous copper: A summary of micro-alloying of Au-group and Pt-group elements. Mater. Trans..

[66] Dan Z., Qin F., Sugawara Y., Muto I., Hara N. (2012). Fabrication of ultrafine nanoporous copper by the minor addition of gold. Mater. Trans..

[67] Dan Z., Qin F., Sugawara Y., Muto I., Hara N. (2013). Elaboration of nanoporous copper by modifying surface diffusivity by the minor addition of gold. Microporous Mesoporous Mater..

[68] Dan Z.H., Qin F.X., Hara N. (2014). Polyvinylpyrrolidone macromolecules function as a diffusion barrier during dealloying. Mater. Chem. Phys..

[69] Xu Q. (2013). Nanoporous Materials: Synthesis and Applications.

[70] Liu D., Yang Z., Wang P., Li F., Wang D., He D. (2013). Preparation of 3D nanoporous copper-supported cuprous oxide for high-performance lithium ion battery anodes. Nanoscale.

[71] Xu C., Wang R., Zhang Y., Ding Y. (2010). A general corrosion route to nanostructured metal oxides. Nanoscale.

[72] Hao Q., Li M., Jia S., Zhao X., Xu C. (2013). Controllable preparation of Co_3_O_4_ nanosheets and their electrochemical performance for Li-ion batteries. RSC Adv..

[73] Jia S., Song T., Zhao B., Zhai Q., Gao Y. (2014). Regular Fe_3_O_4_ octahedrons with excellent soft magnetic properties prepared by dealloying technique. J. Alloy. Compd..

[74] Liu Z., Yamazaki T., Shen Y., Meng D., Kikuta T., Nakatani N., Kawabata T. (2008). Dealloying derived synthesis of W nanopetal films and their transformation into WO_3_. J. Phys. Chem. C.

[75] Zhao Z., Xu J., Liaw P., Wu B., Wang Y. (2014). One-step formation and photocatalytic performance of spindle-like TiO_2_ nanorods synthesized by dealloying amorphous Cu_50_Ti_50_ alloy. Corros. Sci..

[76] Qi Z., Gong Y., Zhang C., Xu J., Wang X., Zhao C., Ji H., Zhang Z. (2011). Fabrication and characterization of magnetic nanoporous Cu/(Fe,Cu)_3_O_4_ composites with excellent electrical conductivity by one-step dealloying. J. Mater. Chem..

[77] Wang H., Jiang M., Su J., Liu Y. (2014). Fabrication of porous CuO nanoplate-films by oxidation-assisted dealloying method. Surf. Coat. Technol..

[78] Zhang B., Chen Y., Guo H. (2014). Electrochemical behavior of Cu–Hf–Al amorphous films. ECS Electrochem. Lett..

[79] Zahrani E., Alfantazi A. (2012). Molten salt induced corrosion of Inconel 625 superalloy in PbSO_4_–Pb_3_O_4_–PbCl_2_–Fe_2_O_3_–ZnO environment. Corros. Sci..

[80] Wang Z. (2013). Fabrication of Nanoporous Copper and Nano/Micro Cuprous Oxide Particles by Dealloying Method.

[81] Chen K., Xue D. (2014). Cu-based materials as high-performance electrodes toward electrochemical energy storage. Funct. Mater. Lett..

[82] Poizot P., Laruelle S., Grugeon S., Dupont L., Tarascon J. (2000). Nano-sized transition-metal oxides as negative-electrode materials for lithium-ion batteries. Nature.

[83] Xu H., Wang W., Zhu W. (2006). Shape evolution and size-controllable synthesis of Cu_2_O octahedra and their morphology-dependent photocatalytic properties. J. Phys. Chem. B.

[84] Qiu H., Lu L., Huang X., Zhang Z., Qu Y. (2010). Immobilization of horseradish peroxidase on nanoporous copper and its potential applications. Bioresour. Technol..

[85] Jia F., Zhao J., Yu X. (2013). Nanoporous Cu film/Cu plate with superior catalytic performance toward electro-oxidation of hydrazine. J. Power Sources.

[86] Luo X., Li R., Zong J., Zhang Y., Li H., Zhang T. (2014). Enhanced degradation of azo dye by nanoporous-copper-decorated Mg–Cu–Y metallic glass powder through dealloying pretreatment. Appl. Surf. Sci..

[87] Chen L., Fujita T., Ding Y., Chen M. (2010). A three-dimensional gold-decorated nanoporous copper core–shell composite for electrocatalysis and nonenzymatic biosensing. Adv. Funct. Mater..

[88] Zhang S., Zheng Y., Yuan L., Zhao L. (2014). Ni–B amorphous alloy nanoparticles modified nanoporous Cu toward ethanol oxidation in alkaline medium. J. Power Sources.

[89] Li M., Su Y., Zhao J., Geng H., Zhang J., Zhang L., Yang C., Zhang Y. (2015). One-pot preparation of thin nanoporous copper foils with enhanced light absorption and SERS properties. CrystEngComm.

[90] Chen L., Zhang L., Fujita T., Chen M. (2009). Surface-enhanced Raman scattering of silver@nanoporous copper core–shell composites synthesized by an *in situ* sacrificial template approach. J. Phys. Chem. C.

[91] Xu C., Liu Y., Wang J., Geng H., Qiu H. (2011). Fabrication of nanoporous Cu–Pt(Pd) core/shell structure by galvanic replacement and its application in electrocatalysis. ACS Appl. Mater. Interfaces.

[92] Zhang S., Zheng Y., Yuan L., Wang X., Zhao L. (2014). In situ synthesis of nickel–boron amorphous alloy nanoparticles electrode on nanoporous copper film/brass plate for ethanol electro-oxidation. Int. J. Hydrog. Energy.

[93] Kou T., Si C., Gao Y., Frenzel J., Wang H., Yan X., Bai Q., Eggeler G., Zhang Z. (2014). Large-scale synthesis and catalytic activity of nanoporous Cu–O system towards CO oxidation. RSC Adv..

[94] Kou T., Wang Y., Zhang C., Sun J., Zhang Z. (2013). Adsorption behavior of methyl orange onto nanoporous core–shell Cu@Cu_2_O nanocomposite. Chem. Eng. J..

[95] Li G., Song Y., Zhang L., Wei X., Song X., Sun Z. (2013). Nanoporous copper silicon composite prepared by chemical dealloying as anode material for Lithium-ion batteries. Funct. Mater. Lett..

[96] Hou C., Lang X., Han G., Li Y., Zhao L., Wen Z., Zhu Y., Zhao M., Li J., Lian J. (2013). Integrated solid/nanoporous copper/oxide hybrid bulk electrodes for high-performance lithium-ion batteries. Sci. Rep..

[97] Winter M., Brodd R. (2004). What are batteries, fuel cells, and supercapacitors?. Chem. Rev..

[98] Qiu H., Xu H., Liu L., Wang Y. (2015). Correlation of the structure and applications of dealloyed nanoporous metals in catalysis and energy conversion/storage. Nanoscale.

[99] Simon P., Gogotsi Y. (2008). Materials for electrochemical capacitors. Nat. Mater..

[100] Miller J., Simon P. (2008). Electrochemical capacitors for energy management. Science.

[101] Huang H., Zhang W., Fu Y., Wang X. (2015). Controlled growth of nanostructured MnO_2_ on carbon nanotubes for high-performance electrochemical capacitors. Electrochim. Acta.

[102] Miller J., Outlaw R., Holloway B. (2011). Graphene electric double layer capacitor with ultra-high-power performance. Electrochim. Acta.

[103] Guo Y., Shi Z., Chen M., Wang C. (2014). Hierarchical porous carbon derived from sulfonated pitch for electrical double layer capacitors. J. Power Sources.

[104] Liu D., Shen J., Liu N., Yang H., Du A. (2013). Preparation of activated carbon aerogels with hierarchically porous structures for electrical double layer capacitors. Electrochim. Acta.

[105] Du X., Zhao W., Wang Y., Wang C., Chen M., Qi T., Hua C., Ma M. (2013). Preparation of activated carbon hollow fibers from ramie at low temperature for electric double-layer capacitor applications. Bioresour. Technol..

[106] Kuratani K., Kiyobayashi T., Kuriyama N. (2009). Influence of the mesoporous structure on capacitance of the RuO_2_ electrode. J. Power Sources.

[107] Cheng S., Yang L., Chen D., Ji X., Jiang Z., Ding D., Liu M. (2014). Phase evolution of an alpha MnO_2_-based electrode for pseudo-capacitors probed by in operando Raman spectroscopy. Nano Energy.

[108] Zhang Y., Li L., Shi S., Xiong Q., Zhao X., Wang X., Gu C., Tu J. (2014). Synthesis of porous Co_3_O_4_ nanoflake array and its temperature behavior as pseudo-capacitor electrode. J. Power Sources.

[109] Yuan C., Hou L., Feng Y., Xiong S., Zhang X. (2013). Sacrificial template synthesis of short mesoporous NiO nanotubes and their application in electrochemical capacitors. Electrochim. Acta.

[110] Bavio M., Acosta G., Kessler T. (2014). Polyaniline and polyaniline–carbon black nanostructures as electrochemical capacitor electrode materials. Int. J. Hydrog. Energy.

[111] Lee H., Cho M., Kim I., Nam J., Lee Y. (2010). RuO*_x_*/polypyrrole nanocomposite electrode for electrochemical capacitors. Synth. Met..

[112] Aradilla D., Estrany F., Casellas F., Iribarren J., Alemán C. (2014). All-polythiophene rechargeable batteries. Org. Electron..

[113] Cortie M., Maaroof A., Smith G. (2005). Electrochemical capacitance of mesoporous gold. Gold Bull..

[114] Snyder J., Asanithi P., Dalton A., Erlebacher J. (2008). Stabilized nanoporous metals by dealloying ternary alloy precursors. Adv. Mater..

[115] Lang X., Hirata A., Fujita T., Chen M. (2011). Nanoporous metal/oxide hybrid electrodes for electrochemical supercapacitors. Nat. Nanotechnol..

[116] Lang X., Zhang L., Fujita T., Ding Y., Chen M. (2012). Three-dimensional bicontinuous nanoporous Au/polyaniline hybrid films for high-performance electrochemical supercapacitors. J. Power Sources.

[117] Meng F., Ding Y. (2011). Sub-micrometer-thick all-solid-state supercapacitors with high power and energy densities. Adv. Mater..

[118] Wang L., Balka T. (2014). Synthesis of nanoporous nickel thin films from various precursors. Philos. Mag. Lett..

[119] Dan Z., Qin F., Sugawara Y., Muto I., Hara N. (2012). Bimodal nanoporous nickel prepared by dealloying Ni_38_Mn_62_ alloys. Intermetallics.

[120] Fukumizu T., Kotani F., Yoshida A., Katagiri A. (2006). Electrochemical formation of porous nickel in zinc chloride-alkali chloride melts. J. Electrochem. Soc..

[121] Hakamada M., Mabuchi M. (2009). Preparation of nanoporous Ni and Ni–Cu by dealloying of rolled Ni–Mn and Ni–Cu–Mn alloys. J. Alloy. Compd..

[122] Qiu H., Kang J., Liu P., Hirata A., Fujita T., Chen M. (2014). Fabrication of large-scale nanoporous nickel with a tunable pore size for energy storage. J. Power Sources.

[123] Dong C., Bai Q., Cheng G., Zhao B., Wang H., Gao Y., Zhang Z. (2015). Flexible and ultralong-life cuprous oxide microsphere-nanosheets with superior pseudocapacitive properties. RSC Adv..

[124] Dong C., Wang Y., Xu J., Cheng G., Yang W., Kou T., Zhang Z., Ding Y. (2014). 3D binder-free Cu_2_O@Cu nanoneedle arrays for high-performance asymmetric supercapacitors. J. Mater. Chem. A.

[125] Momeni M., Nazari Z., Kazempour A., Hakimiyan M., Mirhoseini S. (2014). Preparation of CuO nanostructures coating on copper as supercapacitor materials. Surf. Eng..

